# Turkish physicians’ approach to lesbian, gay, bisexual, transgender, and other gender and sexual minority individuals and their sexual health

**DOI:** 10.1093/sexmed/qfaf043

**Published:** 2025-06-09

**Authors:** Gökhan Çeker, Ersan Arda, Özer Ural Çakıcı, Murat Gül, Muhammed Arif İbiş, Kerem Gençer Kutman, Rahime Duygu Temeltürk, Tufan Çiçek, İrem Akdemir, Meral Çeker, Özlem Gökçe, Mehmet Hamza Gültekin, Yalçın Kızılkan, Hakan Anıl, Murat Demir, Emre Ünal, Ugur Akgün, Batuhan Turgay, Tolga Muharrem Okutucu, Çagrı Dogan, Harun Bal

**Affiliations:** Department of Urology, Basaksehir Cam and Sakura City Hospital, Istanbul, 34480, Turkey; Department of Histology and Embryology, Hamidiye Institute of Health Sciences, University of Health Sciences, Istanbul, 34668, Turkey; Department of Urology, School of Medicine, Trakya University, Edirne, 22030, Turkey; Department of Urology, Private Practice, Ankara, 06370, Turkey; Department of Andrology, Faculty of Medicine, Selcuk University, Konya, 42150, Turkey; Department of Urology, School of Medicine, Ankara University, Ankara, 06230, Turkey; Department of Urology, Sincan Training and Research Hospital, Ankara, 06930, Turkey; Department of Child and Adolescent Psychiatry, School of Medicine, Ankara University, Ankara, 06620, Turkey; Department of Interdisciplinary Neuroscience, Institute of Health Sciences, Ankara University, Ankara, 06340, Turkey; Autism Intervention and Research Center, Ankara University, Ankara, 06260, Turkey; Department of Urology, Etlik City Hospital, Ankara, 06170, Turkey; Department of Infectious Diseases and Clinical Microbiology, School of Medicine, Ankara University, Ankara, 06230, Turkey; Department of Infectious Diseases and Clinical Microbiology, Esenler Maternity and Child Health Hospital, Istanbul, 34230, Turkey; Department of Infectious Diseases and Clinical Microbiology, Basaksehir Cam and Sakura City Hospital, Istanbul, 34480, Turkey; Department of Urology, Cerrahpasa Faculty of Medicine, Istanbul University-Cerrahpasa, Istanbul, 34098, Turkey; Department of Urology, Ankara Bilkent City Hospital, Ankara, 06800, Turkey; Department of Urology, Faculty of Medicine, Istanbul Aydın University, Istanbul, 34295, Turkey; Department of Urology, Faculty of Medicine, Van Yüzüncü Yıl University, Van, 65120, Turkey; Department of Psychiatry, Etlik City Hospital, University of Health Sciences, Ankara, 06170, Turkey; Department of Urology, Bursa City Hospital, Bursa, 16110, Turkey; Department of Obstetrics and Gynecology, School of Medicine, Ankara University, Ankara, 06620, Turkey; Department of Urology, Private Practice, Istanbul, 34758, Turkey; Department of Urology, Faculty of Medicine, Tekirdag Namik Kemal University, Tekirdag, 59100, Turkey; Department of Urology, Faculty of Medicine, Mugla Sıtkı Kocman University, Mugla, 48000, Turkey

**Keywords:** barriers to care, LGBT persons, LGBTQqueers, sexual health

## Abstract

**Background:**

Lesbian, gay, bisexual, transgender, and other gender and sexual minority (LGBT+) individuals often face healthcare disparities, and physicians’ knowledge, attitudes, and clinical preparedness significantly impact access to competent care.

**Aim:**

This study evaluated Turkish physicians’ perspectives, knowledge, and clinical approaches to LGBT+ sexual health, highlighting educational and clinical gaps.

**Methods:**

A nationwide cross-sectional survey was conducted among physicians from 10 specialties involved in LGBT+ sexual health. The survey, administered anonymously via Google Forms between June 4, 2024, and February 1, 2025, included sociodemographic questions and items assessing attitudes, clinical experience, and guideline familiarity. Statistical analyses included descriptive statistics, chi-square, Fisher’s exact tests, and binary logistic regression to identify predictors of physician attitudes and perceived competence.

**Outcomes:**

The primary outcome was to assess physicians’ perspectives, competency, and willingness to provide LGBT+ healthcare, including gender-affirming procedures.

**Results:**

Among 745 participants, 58.8% considered LGBT+ identities normal, while 22.9% classified them as psychiatric disorders. Perceiving LGBT+ identities as normal was significantly associated with being female (OR = 3.12, 95% CI: 1.96-4.96, *P* < .001), prior experience treating LGBT+ patients (OR = 2.22, 95% CI: 1.60-3.07, *P* < .001), and physician specialty. This view was most common among psychiatrists (*P* = .012) and child and adolescent psychiatrists (*P* = .015). Physicians’ views were primarily shaped by education (43.2%) and socio-cultural environment (40.9%). Although 63.9% had treated LGBT+ patients, only 28.2% felt competent, and 11.5% were aware of relevant guidelines. Only 18% of surgical specialists supported gender-affirming procedures. The most commonly cited reason for reluctance was lack of surgical experience (44.8%), along with concerns related to religious beliefs, absence of a surgical team, and potential surgical complications. Ethical dilemmas were evident, as 58.3% believed LGBT+ patients face discrimination in healthcare, and 21.9% supported a physician’s right to refuse care based on personal beliefs.

**Clinical Implications:**

Enhancing physicians’ education and competency in LGBT+ healthcare through structured training and standardized guidelines is crucial to improving equitable healthcare delivery.

**Strengths and Limitations:**

This study provides novel insights into physicians’ attitudes and practices regarding LGBT+ healthcare in Turkey. However, self-reported data may introduce response bias, and findings may not be fully generalizable to other regions.

**Conclusion:**

Significant educational and clinical gaps persist in LGBT+ healthcare. Addressing these through structured training programs, standardized protocols, and multidisciplinary collaboration is essential to ensuring competent, inclusive, and ethical medical care.

## Introduction

Sexual health encompasses the right to a safe and pleasurable sexual life and is an integral part of overall well-being.^1^ Ensuring equitable healthcare access for all individuals, including lesbian, gay, bisexual, transgender, and other gender and sexual minority (LGBT+) individuals, is a fundamental duty of the medical community. However, research has shown that LGBT+ individuals often face barriers in accessing competent and inclusive healthcare services, leading to disparities in sexual health outcomes.^2^ One of the critical factors contributing to these disparities is the preparedness and perception of healthcare professionals regarding LGBT+ sexual health.

While previous studies have examined the challenges LGBT+ individuals face in accessing healthcare in Turkey,^3^^,^^4^ there is limited research on how physicians from different specialties approach LGBT+ sexual health. This is particularly relevant for surgical disciplines involved in gender-affirming procedures (eg, urology, plastic surgery, obstetrics and gynecology) and clinical specialties that provide sexual health services (eg, psychiatry, endocrinology, infectious diseases). Given that LGBT+ individuals often require multidisciplinary care from admission to follow-up, collaboration among these specialties is essential for ensuring comprehensive and effective healthcare.

With the growing emphasis on inclusive healthcare, assessing Turkish physicians’ perceptions, clinical approaches, and competency regarding LGBT+ sexual health is crucial. Additionally, the availability and use of evidence-based guidelines in this field remain underexplored. Sociocultural beliefs and ethical debates surrounding religious freedom may further complicate clinical practices. To address these issues, we conducted a nationwide survey among physicians from multiple specialties to evaluate their attitudes, clinical practices, and confidence in managing LGBT+ patients, as well as their reliance on clinical guidelines.

Specifically, this study addresses the following research questions:

- How do Turkish physicians perceive and define LGBT+ identities in clinical contexts?
- What are the main factors influencing their attitudes and clinical decisions?
- Do they feel competent and ethically aligned in managing LGBT+ sexual health concerns?
- Do Turkish physicians follow or recognize any LGBT+ healthcare guidelines, given the lack of national protocols?

## Methods

### Ethics approval and participants

This cross-sectional study included specialists from 10 professional groups in Turkey that are actively involved in LGBT+ sexual health: general urology, andrology, psychiatry, sexual psychotherapy, family medicine, obstetrics and gynecology, plastic and reconstructive surgery, child and adolescent psychiatry, endocrinology, and infectious diseases.

Ethical approval was obtained from the Trakya University Faculty of Medicine Non-Interventional Scientific Research Ethics Committee (No: 11/17, 03.06.2024). Informed consent was obtained electronically, and individuals who did not provide consent or complete the survey were excluded. No incentives were provided to the participants.

### Survey development and data collection

To evaluate Turkish physicians’ perspectives, knowledge, and clinical approaches toward LGBT+ sexual health, members of the Andrology Working Group of the Society of Urological Surgery in Turkey (SUST)^5^ were invited to develop a multiple-choice questionnaire. The item generation process initially yielded 58 questions, which were reviewed and refined through expert consensus. A panel of 5 professionals—including psychiatrists, urologists, and an obstetrician/gynecologist—assessed the questionnaire for content validity, thematic clarity, and clinical relevance, aligning items with current literature on attitudes, clinical behavior, and ethical considerations. It was not intended as a psychometric scale but rather as a structured data collection tool. While no formal psychometric validation (eg, Cronbach’s alpha) was performed due to the descriptive and exploratory nature of the study, items were carefully reviewed to eliminate redundancy and thematic overlap. The final version consisted of 33 items, structured to efficiently capture physicians’ clinical insights into LGBT+ sexual health. To maintain brevity and minimize participant fatigue in this large-sample, exploratory study, Likert-scale formats were not used. Instead, the questionnaire included dichotomous (eg, yes/no), multiple-choice, and an open-text item. In terms of response structure, certain questions—such as those assessing factors influencing attitudes—allowed multiple answers to reflect the complexity of influencing variables. In contrast, items evaluating dominant perceptions (eg, whether LGBT+ identities are viewed as normal, psychiatric, or sexual health issues) required a single forced-choice response. An open-text response was used selectively, prompting participants who reported awareness of LGBT-specific healthcare guidelines to specify which guideline(s) they were familiar with. These formats were chosen to promote response completion, reduce ambiguity, and ensure clarity in an anonymous, self-administered online setting. While this approach limited more nuanced statistical analyses, it was deemed appropriate given the survey’s scope, design, and the nature of the questions.

The survey was conducted anonymously using Google Forms, which allowed for efficient and secure data collection, between June 4, 2024, and February 1, 2025. Participants were recruited through email invitations sent to members of the SUST Andrology Working Group and other active members of SUST. The survey link was also disseminated on social media to maximize participation. Due to ethical considerations and to maintain respondent anonymity, IP address tracking was not performed. Although Google Forms does not provide IP address verification, additional measures were taken to ensure data integrity. Duplicate data control was conducted manually: The dataset was carefully screened for identical or suspiciously similar responses, and no duplicate entries were detected. Furthermore, the survey link was distributed via personalized invitation links and through professional medical networks to minimize the possibility of multiple submissions from the same respondent. Participants were also required to confirm their final submission, and incomplete responses were excluded. While technical verification such as IP tracking was not feasible, these precautions were implemented to enhance data reliability and ensure that each response reflected a unique participant.

Participants were first informed about the study’s objective before beginning the survey: to assess Turkish physicians’ perspectives, knowledge, and clinical approaches toward the sexual health of LGBT+ individuals. Respondents had the option to review and modify their responses while completing the survey. The study followed CHERRIES guidelines for online surveys, with compliance details provided in the supplementary file A.^6^

### Measures

The survey consisted of 2 sections: sociodemographic characteristics (8 items) and questions assessing perspectives, knowledge, and clinical approaches to LGBT+ sexual health (25 items). The sociodemographic section included variables such as age, gender, specialty, and professional experience (Table 1).

**Table 1:** Participant demographics and professional characteristics.

**Category**	**Subcategory**	**Number**	**(%)**
**Sex**	Male	498	(66.8)
	Female	242	(32.5)
	Prefer not to say	5	(0.7)
**Age (min = 23, max = 71, mean = 34.92 ± 8.48)**	<30 years	231	(31.0)
	30-39 years	331	(44.0)
	40-49 years	124	(16.7)
	50-59 years	50	(6.7)
	60-69 years	8	(1.1)
	≥70 years	1	(0.1)
**Professional background**	General urology	319	(42.8)
	Obstetrics and gynecology	110	(14.8)
	Psychiatry	66	(8.8)
	Infectious diseases and clinical microbiology	53	(7.1)
	Andrology	43	(5.8)
	Child and adolescent psychiatry	41	(5.5)
	Endocrinology and metabolism	40	(5.4)
	Sexual psychotherapist (family physician/general practitioner)	36	(4.8)
	Plastic, reconstructive, and aesthetic surgery	29	(3.9)
	Sexual psychotherapist (psychiatrist)	8	(1.1)
**Title**	General practitioner	22	(2.9)
	Resident doctor	329	(44.2)
	Specialist doctor	265	(35.6)
	Assistant professor, M.D.	34	(4.5)
	Associate professor, M.D.	66	(8.9)
	Professor, M.D.	29	(3.9)
**Practice setting**	Academic	570	(76.5)
	Private	88	(11.8)
	Public	87	(11.7)
**Years in practice (in specialty):**	<5 years	386	(51.8)
	5-10 years	147	(19.7)
	>10 years	212	(28.5)
**Years in practice related to sexual health**	<5 years	564	(75.7)
	5-10 years	72	(9.7)
	>10 years	109	(14.6)
**Membership in sexual health societies**	Members	225	(30.2)
	Non-members	520	(69.8)

The second section evaluated physicians’ perceptions of LGBT+ individuals, familiarity with relevant guidelines, and attitudes toward clinical and ethical issues such as gender-affirming care and HIV/STI screening (Supplementary file-B). Participants who reported familiarity with LGBT-specific healthcare guidelines were prompted to specify which guideline(s) they were familiar with in an open-text format. Guidelines were considered relevant if they contained dedicated content addressing the health needs of LGBT+ individuals. Examples included the WPATH Standards of Care^7^ and the Endocrine Society Clinical Practice Guidelines,^8^ which provide protocols for gender-affirming care. More general documents—such as the European Association of Urology guidelines—were excluded unless they contained explicit LGBT-specific sections. This item was designed to assess participants’ existing knowledge without providing examples during the survey.

### Data analysis

Statistical analysis was conducted using SPSS (version 27.0.1.0, IBM, Armonk, NY, USA). Descriptive statistics were presented as frequencies (n) and percentages (%). Categorical variables were compared using the chi-square test or Fisher’s exact test, as appropriate. For items allowing multiple responses, the multiple-response analysis module in SPSS was utilized. Furthermore, a binary logistic regression analysis was conducted to determine independent predictors among the candidate explanatory variables. A *P*-value <.05 was considered statistically significant.

## Results

### Participant demographics

A total of 756 individuals were considered for the study; however, 11 did not provide consent, leaving 745 responses for analysis. Their demographic and professional characteristics are detailed in Table 1.

### Perspectives, knowledge, and clinical approaches toward the sexual health of LGBT+ individuals

**Figure 1:**
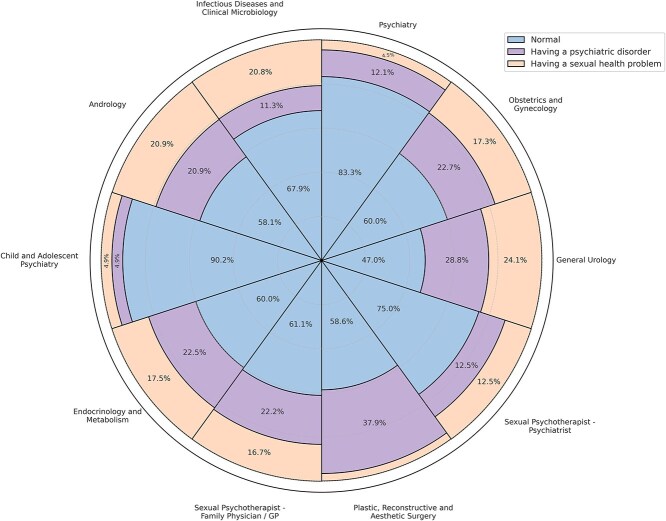
Perspectives of physicians on lesbian, gay, bisexual, transgender, and other gender and sexual minority (LGBT+) individuals by specialty.

Among participants, 58.8% (n = 438) considered LGBT+ individuals completely normal, while 22.9% (n = 171) viewed them as having a psychiatric disorder, and 18.3% (n = 136) perceived them as having sexual health problems. To identify the factors associated with perceiving LGBT+ individuals as “normal,” a binary logistic regression analysis was conducted. Among the examined variables, only gender, specialty, and prior experience in treating LGBT+ patients were found to be statistically significant predictors (*P* < .05). Female physicians were significantly more likely than male physicians to perceive LGBT+ individuals as normal (OR = 3.12, 95% CI: 1.96-4.96, *P* < .001). Psychiatrists (OR = 2.59, 95% CI: 1.24-5.43, *P* = .012) and child and adolescent psychiatrists (OR = 4.07, 95% CI: 1.32-12.58, *P* = .015) were also more likely to hold this view compared to general urologists (Figure 1). Additionally, physicians with prior experience treating LGBT+ patients were significantly more likely to perceive them as normal (OR = 2.22, 95% CI: 1.60-3.07, *P* < .001). However, physicians’ geographic region of practice within Turkey was not found to have a statistically significant association with how they classified LGBT+ individuals (*P* = .168).

**Figure 2:**
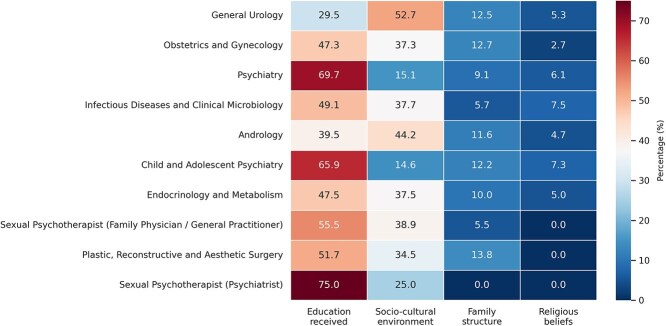
Factors influencing physicians’ perspectives on lesbian, gay, bisexual, transgender, and other gender and sexual minority (LGBT+) individuals.

The primary factors influencing physicians’ views were education (43.2%), socio-cultural environment (40.9%), family structure (11.1%), and religious beliefs (4.7%). Among those influenced by religious beliefs, 68.6% did not consider LGBT+ individuals as completely normal, compared to 39.9% among those influenced by education, family structure, or socio-cultural environment (*P* < .001). Urologists were more influenced by socio-cultural environment (52.7%), while psychiatrists were more influenced by their education (69.7%) (*P* < .001) (Figure 2). The physicians’ geographic region of practice was also significantly associated with the primary factor influencing their views (*P* = .018). In the Marmara (42.5%), Central Anatolia (49.2%), and Eastern Anatolia (50%) regions, education was reported as the most influential factor. In contrast, in the Aegean (56.3%), Mediterranean (50.0%), Black Sea (44.4%), and Southeastern Anatolia (52.6%) regions, the socio-cultural environment was cited as the primary influence.

When asked which specialties LGBT+ individuals should consult for sexual health issues, 22.5% (n = 620/2757) of responses indicated psychiatry (Table 2). Endocrinologists most frequently recommended endocrinology (77.5%), while infectious disease specialists (86.8%) and andrologists (86.0%) most often selected urology. In contrast, physicians from other specialties most frequently selected psychiatry. While 63.9% of physicians had treated an LGBT+ patient, 36.1% had not. A majority (74.9%) believed that a physician should be aware of a patient’s sexual orientation and/or gender identity. However, only 28.2% felt competent in handling LGBT+ sexual health concerns, and only 11.5% were aware of a relevant clinical guideline. To further explore the factors associated with physicians’ self-reported competence in addressing LGBT+ sexual health concerns, a binary logistic regression analysis was performed. The results indicated that physicians who reported receiving adequate training on LGBT+ health issues during residency were significantly more likely to feel competent (OR = 6.62, 95% CI: 3.94-11.13, *P* < .001). Physicians with no prior experience treating LGBT+ individuals were significantly less likely to feel competent in managing LGBT+ sexual health issues (OR = 0.31, 95% CI: 0.20-0.48, *P* < .001). Specialty was also a significant factor. Notably, child and adolescent psychiatrists were more likely to report competence compared to other specialties (OR = 7.09, 95% CI: 1.94-25.93, *P* = .003). Furthermore, physicians with over 11 years of experience in managing sexual dysfunctions were significantly more likely to feel competent in treating LGBT+ patients (OR = 1.79, 95% CI: 1.10-2.93, *P* = .020).

**Table 2:** Distribution of physician responses on which medical specialties should address lesbian, gay, bisexual, transgender, and other gender and sexual minority (LGBT+) sexual health issues.

**Medical specialty**	**Number**	**(%)**
Psychiatry	620	(22.5)
Urology	588	(21.3)
Obstetrics and gynecology	489	(17.7)
Endocrinology and metabolism	311	(11.3)
Child and adolescent psychiatry	274	(9.9)
Plastic, reconstructive, and aesthetic Surgery	205	(7.4)
Infectious diseases and clinical microbiology	151	(5.5)
Medical genetics	119	(4.3)
Total	2757	(100.0)

Respondents could select multiple specialties they deemed appropriate. Shown as percentage of total responses.

Regarding parental concerns about whether their child’s lesbian, gay, or bisexual identity could be changed, 68.2% of physicians stated that it is a sexual orientation difference and should be referred to urology, obstetrics and gynecology, pediatrics, psychiatry, or child and adolescent psychiatry but that it cannot be changed. Similarly, regarding parental concerns about their child’s transgender identity, 68.5% of physicians attributed it to a discrepancy between biological sex and assigned gender and recommended referral to urology, obstetrics and gynecology, psychiatry/child and adolescent psychiatry, or endocrinology while stating that it cannot be changed.

When an LGBT+ individual, either voluntarily or through parental referral, presents with concerns about gender identity or sexual orientation, if physical examination findings and hormonal evaluation are consistent with their biological sex, 65.8% of physicians referred them to psychiatry or child and adolescent psychiatry, while 24.8% stated that they would not provide additional recommendations if no medical pathology was found. Additionally, 8.5% referred such cases for genetic testing. When participants were asked which medical specialties should perform gender-affirming surgeries for transgender individuals, responses (n = 1963) were distributed as follows: 33.3% for plastic surgery, 32.5% for urology, 26.9% for obstetrics/gynecology, and 7.3% for otorhinolaryngology.

**Figure 3:**
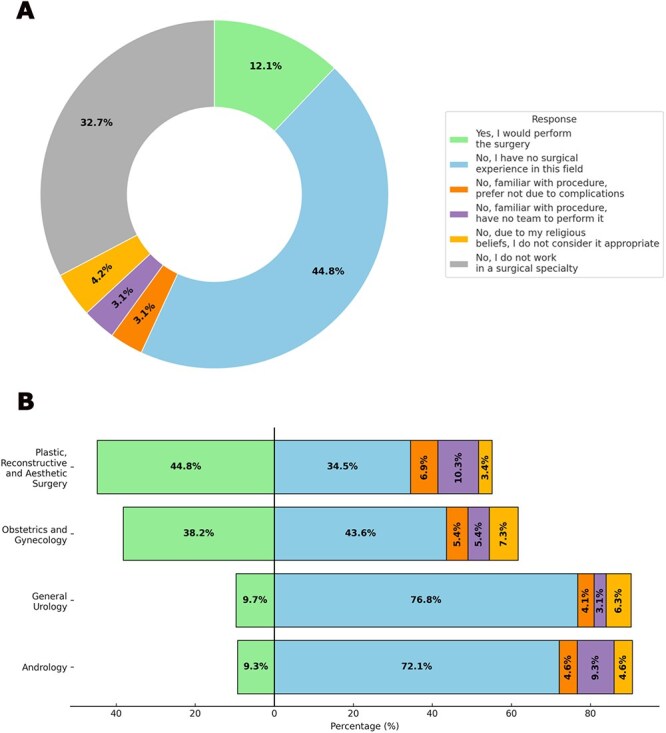
Percentage distribution of responses to the question: “Would you perform gender affirmation surgery if the medico-legal requirements are met?”. (A) Percentage of responses from all physicians. (B) Percentage of responses from physicians in surgical specialties.

Overall, 13.7% of physicians felt adequately trained during residency to assess and manage LGBT+ patients, while 86.3% did not. Furthermore, 69% had no knowledge of the legal processes required for gender-affirming surgery. When asked if they would perform gender-affirming surgery under appropriate medicolegal conditions, only 18% of surgical specialists (12.1% of all participants) responded affirmatively. However, most surgeons refrained from performing the procedure due to various reasons (Figure 3). Urologists were less likely to agree (9.7%) compared to plastic surgeons (44.8%) (*P* < .001).

Overall, 11.9% of participants stated that they were preferred physicians for LGBT+ patients. Among the respondents, 33.3% attributed not being preferred to the tendency of LGBT+ individuals to choose private hospitals or clinics over government institutions. Additionally, 27.9% believed that LGBT+ individuals might select physicians based on recommendations from their communication networks and associations. Meanwhile, 23.9% had no opinion on the matter.

Regarding the establishment of specialized LGBT+ sexual health clinics, 48.2% supported the idea, while others opposed it due to concerns about patient volume or stigma. If proper training were provided, 51.9% expressed willingness to work in such clinics.

**Figure 4:**
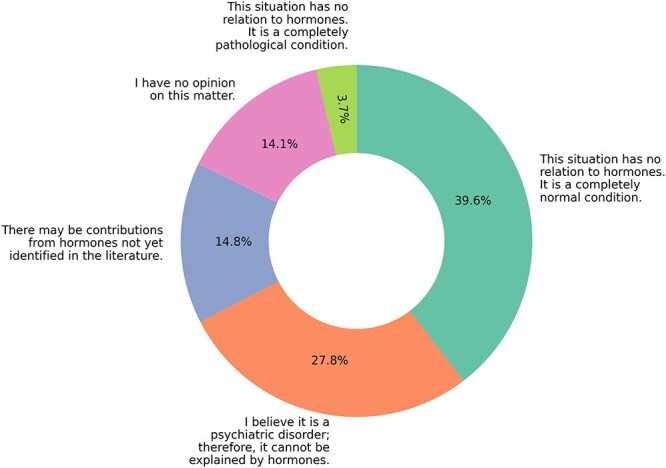
Participants’ perspectives on the hormonal influence on sexual orientation and gender identity.

In terms of LGBT+ identity formation, 48.3% believed sexual orientation and gender identity are innate, while 51.7% attributed them to life experiences and trauma. Furthermore, 71.8% believed that gender identity and sexual orientation could not be genetically identified. Participants were also asked about potential explanations for gender identity and sexual orientation differences in LGBT+ individuals whose hormonal levels fall within the normal range for their biological sex. The distribution of responses is presented in Figure 4.

A majority (58.3%) believed that LGBT+ patients frequently face discrimination in healthcare settings, while 20.3% thought it occurs rarely, 9.9% believed it happens always, and 11.5% did not think discrimination exists. While 78.1% stated that physicians do not have the right to refuse care for LGBT+ patients based on personal beliefs in accordance with medical ethics and the Hippocratic Oath, 21.9% argued that this right should exist, considering religious and moral grounds and the physician’s freedom of religious practice. Additionally, 62.8% believed social media and video platforms encourage LGBT+ identities.

**Figure 5:**
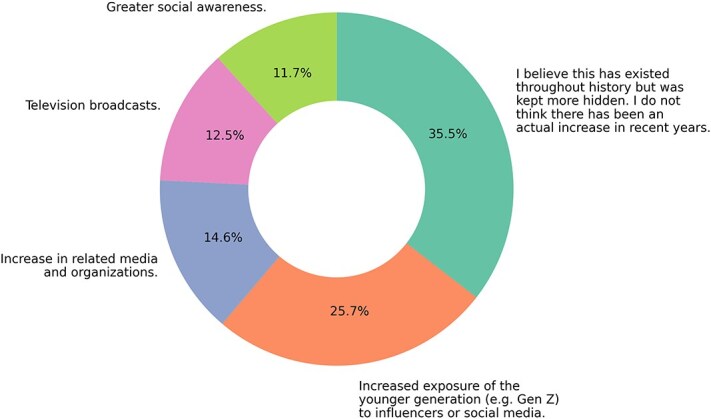
Perceived reasons for the apparent increase in the number of lesbian, gay, bisexual, transgender, and other gender and sexual minority (LGBT+) individuals.

There was no consensus on the perceived increase in the number of LGBT+ individuals in recent years, with varying opinions including greater social awareness, increased media influence, and the belief that this phenomenon has always existed but was previously hidden (Figure 5).

After providing safe sex education and implementing measures to prevent fear of social stigma, 44.7% supported regular HIV/STI testing for LGBT+ individuals at scheduled intervals, 36.9% recommended testing only when a partner was suspected of infection, and 18.4% advocated for mandatory testing.

## Discussion

This study provides valuable insights into Turkish physicians’ attitudes, knowledge, and clinical approaches toward LGBT+ sexual health. Key challenges were identified, including gaps in medical education, disparities in clinical preparedness, and differing perspectives across specialties. Access to LGBT+ healthcare is influenced by medical training, societal norms, and personal beliefs, underscoring the need for systemic change. By identifying existing gaps and variations in clinical attitudes, the study highlights the importance of more inclusive, evidence-based medical practices.

Although a majority of participants recognized LGBT+ identities as normal, a considerable proportion still classified them as psychiatric disorders or sexual health problems. This indicates a persistent gap in medical education and awareness, mirroring findings from other studies where misconceptions about LGBT+ health remain prevalent, even in countries with progressive medical curricula.^9^^,^^10^ The distinction between “psychiatric disorder” and “sexual health problem” may have been unclear for some participants, as the terms were presented separately. This conceptual overlap underscores the need for more precise terminology in future survey designs and highlights the importance of evidence-based medical education, as supported by WHO definitions.^11^

Differences across specialties suggest that physicians’ attitudes toward LGBT+ individuals are shaped by both educational exposure and socio-cultural context. Disciplines with psychosocial training seem more influenced by formal education, while others reflect dominant social norms. Incorporating comprehensive LGBT+ health topics into medical education can enhance understanding and reduce bias among future healthcare providers.^12-14^ In the Turkish context, physicians’ attitudes toward LGBT+ individuals are shaped not only by education and clinical exposure but also by the broader sociopolitical and cultural environment. For instance, Ersoy et al. found that physicians with lower religiosity and less conservative views reported significantly more positive attitudes toward transgender and gender-diverse (TGD) individuals, while political orientation and proximity to TGD persons also influenced their perceptions.^15^ Our findings support this, as physicians influenced by religious beliefs were considerably less likely to view LGBT+ identities as normal, compared to those shaped by education or socio-cultural factors. These results highlight the importance of integrating region-specific social determinants into both medical education and research on LGBT+ health in Turkey. Although religion was cited by only 4.7% of participants, the notable difference in attitudes among this group underscores its potential impact. To respect participant anonymity in a sensitive sociopolitical climate, specific religious affiliations were intentionally not collected.

Despite clinical experience with LGBT+ individuals, most physicians lacked formal training and awareness of relevant guidelines—factors associated with low self-perceived competence and reluctance toward gender-affirming care. These findings align with previous research highlighting the limited exposure of healthcare professionals, medical students, and residents to LGBT+ health and gender-affirming procedures.^16-19^ Only 13.7% of physicians felt adequately trained during residency, and the majority (69%) were unaware of the legal framework for gender-affirming surgery. These gaps underscore the urgent need to integrate structured education on LGBT+ healthcare and its legal aspects into medical training to improve competency and ensure ethical, comprehensive care.^20^

Physicians’ perspectives on the relevance of knowing a patient’s sexual orientation and gender identity were also explored. While 74.9% of participants agreed that this information is important, the aforementioned competency gap may hinder effective communication and discourage LGBT+ individuals from seeking care. Studies have shown that when physicians lack confidence in addressing LGBT+ health needs, it negatively affects patient-provider relationships and leads to poorer health outcomes.^21^ Consequently, many lesbian, gay, and bisexual (LGB) individuals may hesitate to disclose their sexual orientation due to concerns about inadequate care, confidentiality, and discrimination. Without this information, physicians may struggle to provide targeted, patient-centered care that meets the specific health needs of LGB individuals.^16^^,^^22^^,^^23^

A majority of physicians believe LGBT+ patients frequently face discrimination in healthcare. This aligns with global research showing that LGBT+ individuals often avoid care due to fear of mistreatment or inadequate treatment.^24^ Medical disparities continue to affect LGBT+ individuals, even in Western societies where their rights are more widely accepted.^25^ In an Australian study, over half of primary care providers reported discomfort in treating sexual minority patients, largely due to inadequate education on LGBT+ healthcare.^18^ Even LGBT+ physicians have observed discrimination against LGBT+ patients.^26^ These findings highlight the need for structured education to equip healthcare professionals with the knowledge and skills for equitable care. The study underscores the necessity of integrating LGBT+ health training into medical curricula.

Limited research has examined the influence of religion on physicians’ perspectives and their willingness to provide sexual health care to LGBT+ patients. In many countries, laws allow physicians to refuse certain medical services on the grounds that they conflict with their personal beliefs.^27^ Conscientious objection laws have been expanded, particularly in areas such as abortion, fertility treatments, and end-of-life care.^28^

In this study, 21.9% of physicians stated that doctors should have the right to deny treatment to LGBT+ patients based on personal religious or moral convictions. While this remains a contentious ethical issue, in some US states, legislation explicitly protects healthcare providers who refuse care for LGBT+ individuals.^27^ However, such policies significantly hinder LGBT+ patients’ access to medical services and exacerbate existing health disparities.^10^ Given the fundamental responsibilities of the medical profession, there is a strong argument that conscientious objection should be subject to limitations.

This study also highlights the importance of specialized training and policy changes. While 48.2% of participants supported LGBT+ health clinics, concerns about potential stigma and patient volume were raised. However, if proper training were provided, 51.9% expressed willingness to work in such clinics. This underscores the necessity of integrating LGBT+ health topics into mainstream medical education rather than isolating them within niche programs.^29-32^

While the origins of sexual orientation and gender identity are multifactorial, research suggests that biological factors play a significant role.^33-35^ Studies indicate that prenatal hormonal exposure may influence sexual orientation, as seen in research where reduced prenatal testosterone levels were associated with same-sex sexual behavior in animal models.^35^ However, studies have found no significant differences in adult hormone levels between heterosexual, homosexual, and transgender individuals.^36^ Most LGBT+ individuals also report that their identities are innate rather than chosen.^37^ Despite these findings, misconceptions persist among physicians. In this study, nearly half of participants viewed sexual orientation and gender identity as innate, while a similar proportion attributed them to life experiences or trauma, reflecting ongoing ambiguity in clinical perception.

The findings of this study highlight the range of opinions on HIV/STI testing among healthcare professionals after implementing safe sex education and addressing stigma. While 44.7% supported regular screening—consistent with CDC guidelines—over one-third preferred reactive testing based on partner suspicion, and nearly one-fifth supported mandatory testing.^38^ These variations may reflect ongoing gaps in training, unfamiliarity with public health guidelines, or persisting stigmas around sexual health. The support for mandatory testing, in particular, suggests a need for greater emphasis on patient autonomy and ethical standards in clinical decision-making.

Furthermore, the study reveals that most physicians believe social media and video platforms encourage LGBT+ identities. This perception aligns with broader societal debates on media’s influence over gender and sexual identity. While some argue that media representations increase visibility and acceptance, others view them as influential factors in identity development. However, existing research suggests that media primarily provides a space for LGBT+ individuals to access information, representation, and peer support, rather than acting as a determinant of identity formation.^39^^,^^40^

This study has limitations. Self-reported survey data may introduce response bias, as participants might provide socially desirable answers. Additionally, online data collection may have inadvertently excluded healthcare professionals who are less familiar with digital tools, potentially skewing the demographic representation. A further limitation is that certain forced-choice survey items, such as those distinguishing between “psychiatric disorder” and “sexual health problem,” may have restricted participants’ ability to express more nuanced perspectives and introduced minor conceptual overlap, as the distinction between the two may not have been clear to all respondents. Although data on geographic region were collected, the urban versus rural practice setting was not assessed in this study. These factors may play an important role in shaping physician attitudes and should be addressed in future research. Another important limitation is the lack of detailed information regarding the specific clinical guidelines followed by respondents in their medical practice, which could impact the interpretation of their reported approaches. Furthermore, the study did not assess the direct impact of physicians’ knowledge and attitudes on patient outcomes, which remains an essential area for future investigation. Moreover, this study employed purposive, non-random sampling, which may limit the generalizability of findings. However, participants were selected from relevant clinical groups through professional networks, and results should be interpreted within an exploratory framework. Lastly, as the study was conducted among Turkish physicians, the findings may not be fully generalizable to other healthcare systems. Future research should aim to explore these issues in diverse populations and assess the effectiveness of structured educational interventions in improving LGBT+ healthcare competencies among medical professionals.

## Conclusion

This study reveals notable gaps in Turkish physicians’ preparedness to address the sexual health needs of LGBT+ individuals, particularly in areas such as clinical competency, familiarity with guidelines, and comfort with gender-affirming care. Although many physicians expressed openness to inclusive care, a significant proportion still lacked confidence and training. Bridging these gaps requires not only targeted, evidence-based educational interventions but also policy-level strategies to standardize inclusive, competent, and ethical care practices across medical specialties. Future research should explore the effectiveness of structured training programs and assess the sociocultural and institutional factors influencing clinical practice to promote equitable healthcare delivery for LGBT+ populations.

## Supplementary Material

Supplementary_file-A

Supplementary_file-B

## Data Availability

The datasets used and/or analyzed during the current study are available from the corresponding author on reasonable request.
