# Supplementary material for: Turkish physicians’ approach to lesbian, gay, bisexual, transgender, and other gender and sexual minority individuals and their sexual health
Source: Sex Med. 2025 Jun 9;13(3):qfaf043. doi: 10.1093/sexmed/qfaf043 (PMC12147215; doi:10.1093/sexmed/qfaf043)
Supplement: Supplementary_file-A [file supplementary_file-a.pdf]

Supplementary file-A: Checklist for Reporting Results of Internet E-Surveys (CHERRIES)

Item Category	*Checklist Item*	*Explanation*
Design	Describe survey design	The study was a convenience sample study. The survey was created on an online platform (Google Forms). Researchers distributed the survey invitation via email. Participation in the survey was voluntary.
IRB (Institutional Review Board) approval and informed consent process	IRB approval	Trakya University Faculty of Medicine Non-Interventional Scientific Research Ethics Committee, Approval number: 11/17, 03.06.2024 Date: 03.06.2024
	Informed consent	Participants were informed about the survey’s purpose and the principles of anonymity, confidentiality, and voluntary participation before responding. Providing contact information on the form was optional. All personal information was accessible only to the survey administrator.
	Data protection	The collected data was stored on Google’s online servers, with access limited to authorized personnel who had signed a confidentiality agreement to view and analyze the information.
Recruitment process and description of the sample having access to the questionnaire	Open survey versus closed survey	Closed survey – Limited by invitation link.
	Contact mode	Participants were contacted via email, including a link to the survey.
	Advertising the survey	The survey was closed and only available to those invited via email.
Survey administration	Web/E-mail	The survey was published on the “Google Forms” online questionnaire platform.
	Context	The commercial platform specializes in publishing online surveys. This platform was only used for data collection in this study.
	Mandatory/voluntary	Voluntary.
	Incentives	N/A
	Time/Date	The data collection was carried out between June 4, 2024, and February 1, 2025.
	Randomization of items or questionnaires	Randomization was not applied.
	Adaptive questioning	The survey contained only one adaptive question so respondents could skip past unnecessary question based on their answer.
	Number of Items	The survey contained a total number of 33 questions.
	Number of screens (pages)	The survey questions were presented in 4 pages.
	Completeness check	Before the survey was sent, participants were asked to select at least one answer for all questions, ensuring the integrity of the survey.
	Review step	Participants were allowed to navigate back in the survey and change their answers, but no changes were allowed after completing and submitting the survey.
Response rates	Unique site visitor	N/A
	View rate (Ratio of unique survey visitors/unique site visitors)	N/A
	Participation rate (Ratio of unique visitors who agreed to participate / unique first survey page visitors)	Participation in the survey was voluntary. A total of 1900 participants were invited and a total of 745 participated in the survey.
	Completion rate (Ratio of users who finished the survey/users who agreed to participate)	The results were only recorded if all questions were answered, and the participants confirmed the final submission. 756 people took the survey into consideration, 11 people did not give their consent to participate. Therefore, answers from 745 participants were included in the data assessment.
Preventing multiple entries from the same individual	Cookies used	Cookies were not used.
	IP check	N/A
	Log file analysis	N/A
	Registration	N/A
Analysis	Handling of incomplete questionnaires	The results for each participant were only recorded if all questions were answered and the final submission was confirmed.
	Questionnaires submitted with an atypical timestamp	N/A
	Statistical correction	N/A
