# Supplementary material for: Turkish physicians’ approach to lesbian, gay, bisexual, transgender, and other gender and sexual minority individuals and their sexual health
Source: Sex Med. 2025 Jun 9;13(3):qfaf043. doi: 10.1093/sexmed/qfaf043 (PMC12147215; doi:10.1093/sexmed/qfaf043)
Supplement: Supplementary_file-B [file supplementary_file-b.pdf]

Supplemantary file-B. Survey Questions and Responses on Perspectives, Knowledge, and Clinical Approaches to Lesbian, Gay, Bisexual, Transgender, and Other Gender and Sexual Minority (LGBT+) Sexual Health

	Questions	Responses
1	What is your personal opinion about LGBT+ individuals?	- They are completely normal and do not have a disorder.<br>- They have a psychiatric disorder.<br>- They have a sexual health problem.
2	What factors shape your opinion about LGBT+ individuals?	- Education received<br>- Socio-cultural environment<br>- Family structure<br>- Religious beliefs
3	Which medical specialties should LGBT+ individuals consult for sexual health concerns?	- Psychiatry<br>- Urology<br>- Obstetrics and Gynecology<br>- Endocrinology and Metabolism<br>- Child and Adolescent Psychiatry<br>- Plastic, Reconstructive and Aesthetic Surgery<br>- Infectious Diseases and Clinical Microbiology<br>- Medical Genetics
4	Have you ever provided medical care to an LGBT+ patient?	- Yes<br>- No
5	Do you believe that a physician should be aware of an LGBT+ individual's sexual orientation and/or gender identity?	- Yes<br>- No
6	Do you feel competent in addressing the sexual health concerns of LGBT+ individuals?	- Yes<br>- No
7	Is there a guideline you use or can use regarding the treatment of LGBT+ individuals?	- Yes (Please explain):<br>- No<br>- I have no idea

8	How do you approach a parent concerned about their child's lesbian, gay, or bisexual identity and asking if it can be changed?	- I explain that this is a disorder related to sexual orientation and recommend medical and psychiatric evaluation, stating that after treatment, they will become a person like the majority of society.<br><br>- I explain that this is a variation in sexual orientation and recommend referral to relevant specialties, emphasizing that being lesbian, gay, or bisexual cannot be changed.
9	How do you approach a parent concerned about their child identifying as transgender and asking if it can be changed?	- I explain that this results from a discrepancy between biological sex and assigned gender. I recommend referral to urology, gynecology, psychiatry, and endocrinology, emphasizing that they will adapt to the majority of society after treatment.<br><br>- I explain that this results from a discrepancy between biological sex and assigned gender and recommend referral to urology, gynecology, psychiatry, and endocrinology. However, I emphasize that being transgender cannot be changed.
10	If an LGBT+ individual consults you, either voluntarily or through parental referral, due to gender identity or sexual orientation concerns, and their physical examination findings and hormonal evaluation fully align with their apparent biological sex, what would be the next step?	- I state that there is no pathology and do not provide additional recommendations.<br>- I refer them to psychiatry or child and adolescent psychiatry.<br>- I refer them to medical genetics for genetic testing.
11	Which medical specialties should perform gender-affirming surgeries for transgender individuals?	- Urology<br>- Obstetrics and Gynecology<br>- Plastic, Reconstructive and Aesthetic Surgery<br>- Otorhinolaryngology
12	During your residency, did you receive adequate training on the healthcare needs of LGBT+ individuals?	- Yes<br>- No
13	Do you have knowledge about the legal process for gender-affirming surgery in Turkey?	- Yes<br>- No

14	If a transgender individual applies to you for gender-affirming surgery and all the necessary medicolegal requirements are met, would you perform the surgery?	- Yes, I would perform the surgery.<br>- No, I would not perform the surgery; I have no surgical experience in this field.<br>- No, I would not perform the surgery; although I am familiar with the surgical procedures, I prefer not to due to the risk of complications.<br>- No, I would not perform the surgery; although I am familiar with the surgical procedures, I do not have a team to perform it with.<br>- No, I would not perform the surgery; due to my religious beliefs, I do not find aesthetic operations appropriate.<br>- No, I would not perform the surgery; I do not work in a surgical specialty.
15	If you think you have not been a preferred physician for LGBT+ individuals so far, what do you think could be the reason for this?	- I have no idea.<br>- I think LGBT+ individuals may have chosen physicians recommended through their own communication networks and organizations when searching for a doctor.<br>- Since I work in a public institution, I believe these patients may prefer private hospitals or clinics.<br>- I am a physician preferred by LGBT+ patients.
16	How do you think it would be if a clinic dedicated to LGBT+ sexual health were established and these patients were referred there?	- It would be very appropriate.<br>- It would be wrong.<br>- I think such clinics would be unnecessary because I believe the number of patients is low.
17	Would you like to work in a clinic specializing in LGBT+ sexual health if training were provided?	- Yes<br>- No
18	How can we explain the current status of LGBT+ individuals whose hormonal levels are within the normal range for their biological sex in relation to their gender identity/sexual orientation differences?	- This situation is not related to hormones. It is a completely normal condition.<br>- This situation is not related to hormones. It is a completely pathological condition.<br>- There may be a contribution from hormones that have not yet been identified in the literature.<br>- I believe it is a psychiatric disorder, which is why it cannot be explained

		by hormones.<br>- I have no idea.
19	Do you think sexual orientation and gender identity are primarily innate, or do experiences, life events, and traumas play a significant role?	- Innate<br>- Influenced by life experiences and trauma
20	Do you think it is possible to genetically determine gender identity and sexual orientation differences?	- Yes, just as many normal conditions and pathologies can be genetically identified, I believe this condition can also be genetically determined since it exists from birth.<br>- No, I believe these are primarily psychological processes, and aside from the naturally occurring male and female sexes and heterosexual orientation, there is no genetic basis.
21	Do you think LGBT+ individuals face discrimination when seeking medical care for their sexual health?	- Never<br>- Rarely<br>- Frequently<br>- Always
22	Do you believe that physicians have the right to refuse service or treatment for the sexual health of LGBT+ patients?	- I believe that, within the framework of medical ethics and the Hippocratic Oath, physicians do not have such a right.<br>- I believe that, considering the physician's freedom of religious practice, doctors have the right to refuse service or treatment based on religious or moral reasons.
23	Do you think social media and video platforms influence or promote LGBT+ identities?	- Yes<br>- No
24	What do you think is the reason(s) behind the relative increase in the number of LGBT+ individuals in recent years?	- The younger generation/Generation Z being highly exposed to influencers or social media.<br>- Television broadcasts<br>- Increased social awareness<br>- The increase in relevant media and organizations<br>- I believe this has existed throughout history but was more concealed. I don’t think there has been an increase in recent years.

25	It is well known that LGBT+ individuals, especially gay and transgender people, are at a higher risk of HIV and other sexually transmitted infections (STIs). After providing comprehensive safe sex education to protect these individuals from such infections and implementing necessary measures to reduce social stigma, which of the following do you think is the most appropriate approach to take?	- Mandatory routine testing<br>- Recommended routine testing<br>- Testing only if the partner is suspected of having HIV/STIs
